# Insights on spin polarization through the spin density source function†
†Electronic supplementary information (ESI) available: Details on computational methods and on the developed source codes (ESI_1); numerical accuracy of the spin density reconstruction (ESI_2); 3D contour plots of magnetic natural orbital densities and of total spin density [CASSCF(8,8) model wavefunction] (Fig. S1–S3); maps of *s*(**r**) and ∇^2^*s*(**r**) for the magnetic natural orbitals [CASSCF(8,8) model wavefunction] (Fig. S4 and S5) and of ∇^2^*ρ*(**r**), *s*(**r**) and ∇^2^*s*(**r**) for the non-magnetic natural orbitals in the *xz* and *yz* planes for the ^3^B_1_ water molecule [CASSCF(8,8), UHF spin contamination annihilated and not annihilated model wavefunctions] (Fig. S6 and S7); SF and SF_S_ percentage contributions at some reference points (rps) for ^3^B_1_ H_2_O at the UHF/UHF spin contamination annihilated and at the ROHF levels (Fig. S8 and S9). See DOI: 10.1039/c4sc03988b


**DOI:** 10.1039/c4sc03988b

**Published:** 2015-04-14

**Authors:** Carlo Gatti, Ahmed M. Orlando, Leonardo Lo Presti

**Affiliations:** a CNR-ISTM, Istituto di Scienze e Tecnologie Molecolari , Via Golgi 19 , 20133 Milano , Italy . Email: c.gatti@istm.cnr.it; b Center for Materials Crystallography , Aarhus University , Langelandsgade 140 , 8000 Aarhus , Denmark; c Dipartimento di Chimica , Università degli Studi di Milano , Via Golgi 19 , 20133 Milano , Italy

## Abstract

Understanding how spin information is transmitted from paramagnetic to non-magnetic centers is crucial in advanced materials research and calls for novel interpretive tools. Herein, we show that the spin density at a point may be seen as determined by a local source function for such density, operating at all other points of space. Integration of the local source over Bader's quantum atoms measures their contribution in determining the spin polarization at any system's location. Each contribution may be then conveniently decomposed in a *magnetic* term due to the magnetic natural orbital(s) density and in a *reaction* or *relaxation* term due to the remaining orbitals density. A simple test case, ^3^B_1_ water, is chosen to exemplify whether an atom or group of atoms concur or oppose the paramagnetic center in determining a given local spin polarization. Discriminating *magnetic* from *reaction* or *relaxation* contributions to such behaviour strongly enhances chemical insight, though care needs to be paid to the large sensitivity of the latter contributions to the level of the computational approach and to the difficulty of singling out the magnetic orbitals in the case of highly correlated systems. Comparison of source function atomic contributions to the spin density with those reconstructing the electron density at a system's position, enlightens how the mechanisms which determine the two densities may in general differ and how diverse may be the role played by each system's atom in determining each of the two densities. These mechanisms reflect the quite diverse portraits of the electron density and electron spin density Laplacians, hence the different local concentration/dilution of the total and (α–β) electron densities throughout the system. Being defined in terms of an observable, the source function for the spin density is also potentially amenable to experimental determination, as customarily performed for its electron density analogue.

## Abstract 2

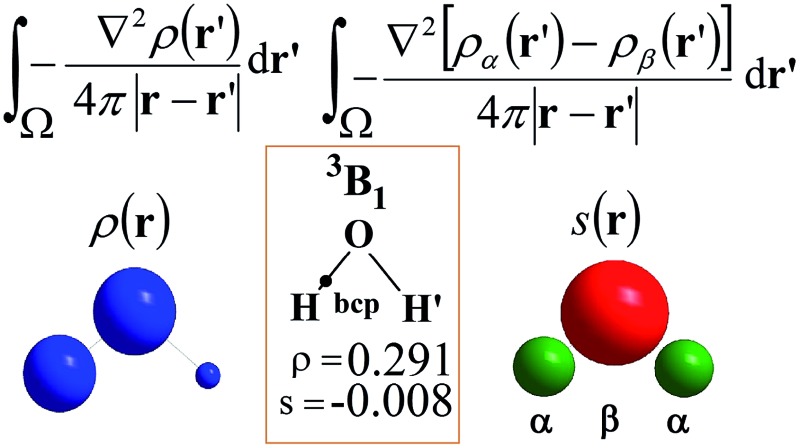
The source function for the spin density *s*(**r**) is introduced, allowing the H and O influence on *s*(**r**) to be disentangled.

## Introduction

Magnetic networks based on molecular and metalorganic paramagnetic species are attractive in several cutting-edge research areas, including advanced sensing,^1^ porous molecular sieves,^2^,^3^ and spintronics.^4^ It is known that pairing among different paramagnetic centers can be exploited through different mechanisms,^5^ often in competition with each other, such as direct exchange, ligand-mediated exchange, superexchange, and so on. The magnetic properties ultimately depend on how the spin information is propagated from a given paramagnetic centre to its neighbouring atoms. In other words, magnetism is inherently due to *non-local* effects, which can be exploited through space or chemical bonds as well.

To achieve a first-principles understanding of magnetism in complex systems the spin density distribution, SDD (*s*(**r**)), is often analyzed. This scalar field is defined as:

1 *s*(**r**) = *ρ*_α_(**r**) – *ρ*_β_(**r**)

with *ρ*_α_(**r**) and *ρ*_β_(**r**) being the spin α and β contributions to the total electron density. Usually, SDD is derived from quantum mechanical simulations, but in principle it is also experimentally accessible through magnetic scattering of polarized X-rays and neutrons.^6^,^7^ A recent split–spin version of the well-known Hansen and Coppens multipolar model^8^ has enabled a joint refinement of X-ray and polarized neutron diffraction data,^9^,^10^ leading to much improved experimental SDD and to first spin-resolved electron density distributions (EDDs). In tandem with the increased availability of large scale facilities providing intense neutron and synchrotron X-ray sources, such a modelling extension will set SDD as a more and more valuable tool to understand and design specific magnetic interactions in complex solid-state networks.^9^–^11^ However, the *s*(**r**) scalar field alone is neither able to provide direct information on the reasons underlying possible spin polarization effects, nor to disentangle the underlying exchange/pairing mechanisms. Interpretive models, generally based on atomic or molecular orbitals considerations, are often used for this purpose.

In this work, a novel SDD-based real-space descriptor is introduced, the spin density Source Function (SF_S_), able to gain, in terms of a cause–effect relationship, quantitative insights on the relative capability of different atoms or groups of atoms in a system to determine the spin density at a given system's location.

## Theory

### The electron density source function

Almost 20 years ago, Bader and Gatti^12^ demonstrated that the electron density at any point **r**, *ρ*(**r**), can be seen as due to a Local Source (LS) function operating at all other points **r′** in space, according to eqn (2) and (3):2
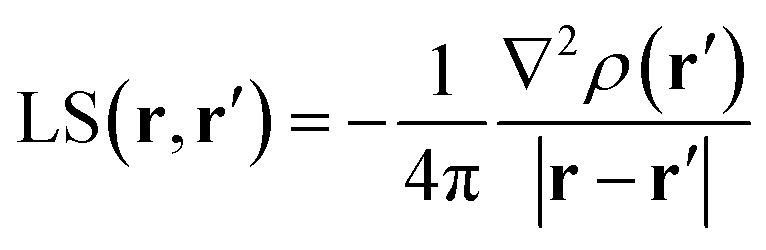

3
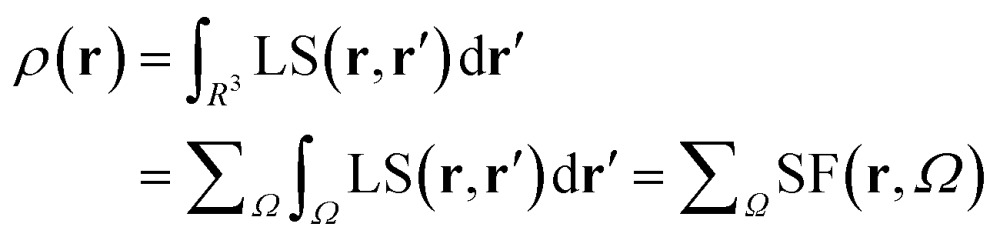



The factor (4π|**r** – **r′**|)^–1^ (eqn (2)) is a Green's function and represents the effectiveness of how the local *cause*, ∇^2^*ρ*(**r′**), contributes to give rise to the *effect*, *ρ*(**r**). Using Bader's recipe^13^ to exhaustively partition the whole space into disjoint quantum atomic basins *Ω*'s, *ρ*(**r**) can be equated to a sum of integral atomic Source Function (SF) contributions (eqn (3)). Such a decomposition scheme highlights that the *ρ*(**r**) field is at any point inherently *not local* in nature, but rather determined by the influence, be it small or relevant, of all other points in the system. It was also shown that eqn (3) enables one to view chemical bonding and other chemical paradigms from a totally new perspective and using only information from the electron density observable and its derivatives. Since the seminal work by Bader and Gatti, the SF descriptor has been extensively and successfully applied to study non-local bonding effects in molecules and crystals,^14^–^23^ using *ab initio* and experimentally derived EDD.

### The spin density source function

It is now straightforward to extend the SF decomposition scheme to the SDD *s*(**r**):4
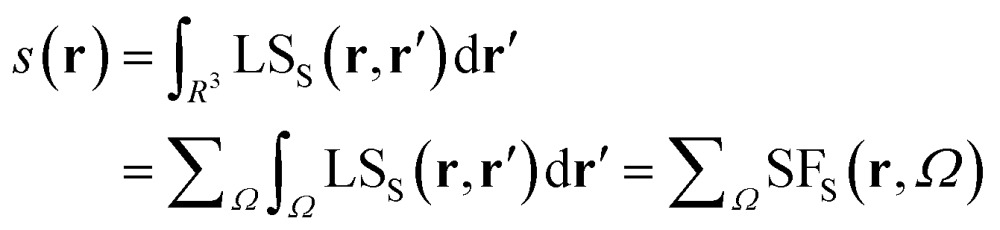



Again, the integral over the whole space is partitioned into contributions from Bader's quantum atoms *Ω*'s, *i.e.* from regions of space bounded by zero-flux surfaces in the **∇***ρ*(**r**) vector field.^13^ As a consequence, ∇^2^*s*(**r′**) does not necessarily sum to zero when integrated over a basin *Ω*. Moreover, at variance with eqn (2), the Local Source LS_S_ is now defined in terms of the Laplacian of the spin density:5
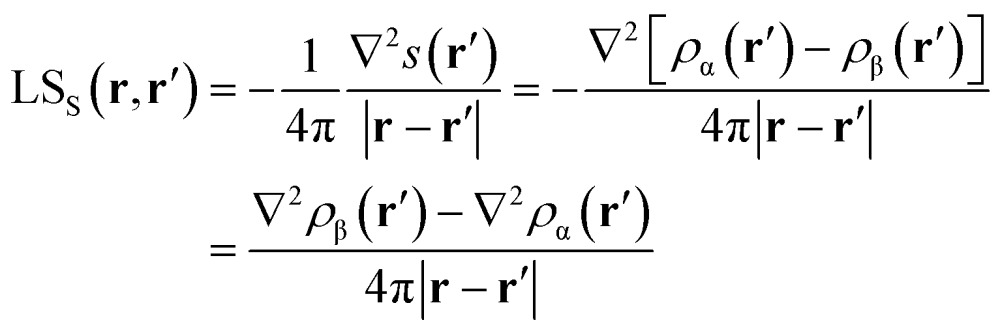



Note that the Green function (4π|**r** – **r′**|)^–1^ is exactly the same as in eqn (2), as it represents a pure geometrical (effectiveness) factor, while the spin density *s* replaces *ρ* in both the local *cause*, ∇^2^*s*(**r′**), and *effect*, *s*(**r**), expressions. This implies that SF and SF_S_ descriptors will generally convey different pictures, reflecting the quite different ways the electron and electron spin densities condense (∇^2^*u*(**r′**) < 0, *u* = *s* or *ρ*) or dilute (∇^2^*u*(**r′**) > 0) themselves throughout the system.

## The spin density Laplacian and local source function

Before applying the SF_S_ descriptor and comparing results with those from the standard SF analysis, it is worth examining in some more detail the intriguing relationships between the Laplacian of the spin density, its α and β components and the ensuing local source function for the spin density.

### A test case: ^3^B_1_ water

**Fig. 1:**
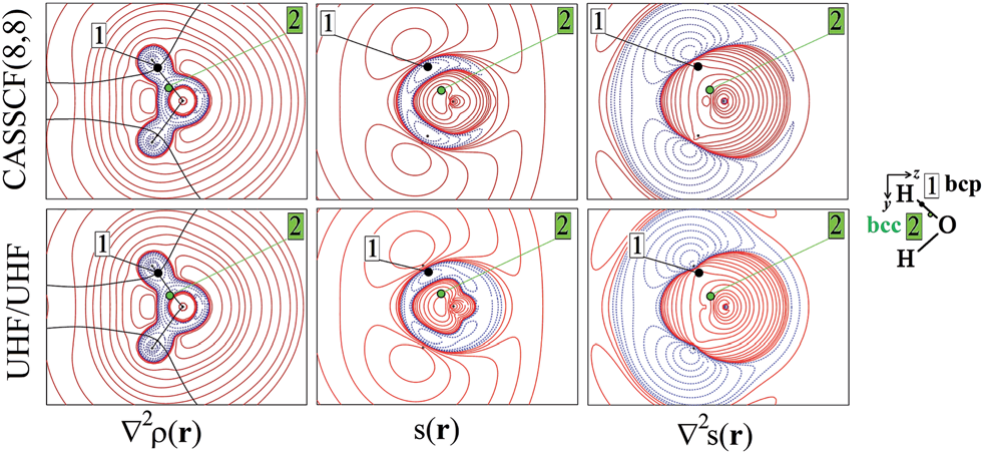
Electron density Laplacian, electron spin density *s*(**r**) and its Laplacian in the (*y*,*z*) plane for ^3^B_1_ H_2_O, at (top) CASSCF(8,8) and (bottom) UHF/UHF spin-contamination annihilated computational levels. Atomic units (a.u.) are used throughout. Contour maps are drawn at intervals of ±(2,4,8) × 10^*n*^, –4 ≤ *n* ≤ 0 (*s*, ∇^2^*s*) and –3 ≤ *n* ≤ 0 (∇^2^*ρ*). Dotted blue (full red) lines indicate negative (positive) values and full black lines mark boundaries of atomic basins. The O–H bond critical point (bcp, 1) and the bonded charge concentration point (bcc, 2) are shown as black and green dots.

**Fig. 2:**
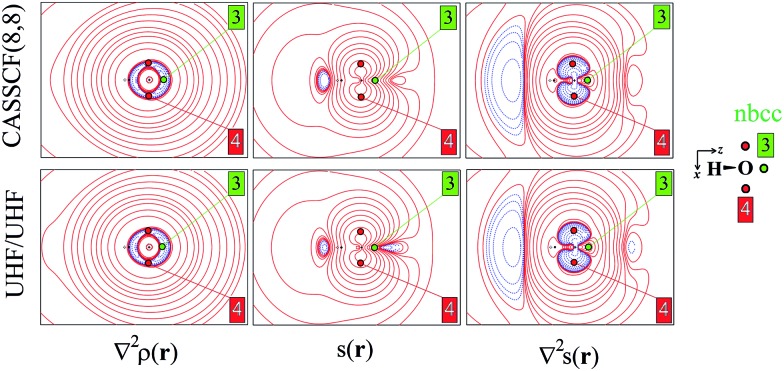
Electron density Laplacian, spin density and its Laplacian in the (*x*,*z*) plane, at (top) CASSCF(8,8) and (bottom) UHF/UHF spin contamination annihilated computational levels. Contour levels as in Fig. 1. The non-bonded charge concentration (nbcc, 3) and the (3,+1) *L*(**r**) rcps (4) are shown as green and red dots.

As an aid for exemplifying this issue, we explore a relatively simple case, *i.e.* water in its ^3^B_1_ state (Fig. 1 and 2) with the nuclei in the (*y*,*z*) plane and the two unpaired electrons lying into two singly occupied orbitals dominated by O p_*x*_-type and O s, p_*z*_-type functions, respectively. Details on the applied levels of theory (CASSCF(8,8), UHF, ROHF all with a 6-311++G(2d,2p) basis set), as well as on the newly developed source codes, are reported in the ESI (ESI_1†).

Besides the (3,–1) bond critical point (bcp) of the *ρ*(**r**) distribution (1 in Fig. 1), we selected some critical points of the –∇^2^*ρ*(**r**) = *L*(**r**) field as suitable references points (rps) for the SF analysis. Namely, (i) the (3,–3) *L*(**r**) maxima along the O–H bonds (2 in Fig. 1) and the maximum in the region of the O lone pair (3 in Fig. 2), as representative of bonded and non-bonded charge concentrations (bcc and nbcc), and (ii) two symmetry-equivalent (3,+1) *L*(**r**) ring points (4 in Fig. 2) lying in the *xz* plane, almost above and below the O atom and associable to unpaired electrons. Table 1 lists the values of *ρ*(**r**), *ρ*_α_(**r**), *ρ*_β_(**r**), *s*(**r**) and of their corresponding Laplacians at these rps, for the three adopted levels of theory and using as a common geometry the UHF/6-311++G(2d,2p) optimized geometry of the spin-contamination annihilated wavefunction. Rps locations correspond to the selected critical points (CPs) for the various wavefunctions, but being CPs of the electron density they almost coincide for the three computational levels.

**Table 1:** Electron density, electron spin density and corresponding Laplacian values (in a.u.) as evaluated at the 1–4 reference points shown in Fig. 1 and 2, for the three adopted computational levels^*a*^ ^*b*^

RP	*ρ*	∇^2^*ρ*	*s*	∇^2^*s*	*ρ*_α_	∇^2^*ρ*_α_	*ρ*_β_	∇^2^*ρ*_β_
**CASSCF(8,8)//UHF(6-311++G(2d,2p))**								
1	0.291	–2.06	–0.0075 (0.0020)	0.24 (0.13)	0.142	–0.91	0.149	–1.15
2	0.888	–5.08	0.0763 (0.0508)	0.90 (1.21)	0.482	–2.09	0.406	–2.99
3	1.022	–6.64	0.0219 (0.0038)	1.73 (1.97)	0.522	–2.46	0.500	–4.18
4	0.614	–1.23	0.3824 (0.3722)	–4.45 (–4.40)	0.498	–2.84	0.116	+1.61

**UHF/(6-311++G(2d,2p)) spin-contamination annihilated wavefunction**								
1	0.288	–2.14	–0.0050 (0.0029)	0.21 (0.11)	0.141	–0.96	0.146	–1.18
2	0.888	–5.17	0.0631 (0.0511)	1.07 (1.18)	0.475	–2.05	0.412	–3.12
3	1.030	–6.85	0.0051 (0.0037)	2.04 (1.95)	0.518	–2.40	0.513	–4.45
4	0.610	–1.18	0.3818 (0.3677)	–4.54 (–4.34)	0.496	–2.86	0.114	+1.68

**ROHF//UHF(6-311++G(2d,2p))**								
1	0.287	–2.14	0.0031	0.11	0.145	–1.01	0.142	–1.13
2	0.890	–5.21	0.0483	1.20	0.469	–2.01	0.421	–3.20
3	1.031	–6.87	0.0032	1.95	0.517	–2.46	0.514	–4.41
4	0.607	–1.13	0.3637	–4.28	0.485	–2.70	0.121	+1.57

^*a*^In parentheses the contributions from the two magnetic orbitals. Note that for the latter *ρ* ≡ *s*, ∇^2^*ρ* ≡ ∇^2^*s*, *ρ*_α_ ≡ *s*, ∇^2^*ρ*_α_ ≡ ∇^2^*s* while *ρ*_β_ and ∇^2^*ρ*_β_ are both null.

^*b*^For the ROHF wavefunction, *s* ≡ *ρ*_α,mag_ and ∇^2^*s* ≡ ∇^2^*ρ*_α,mag_ where *ρ*_α,mag_ and ∇^2^*ρ*_α,mag_ denote the magnetic contribution to *ρ*_α_ and ∇^2^*ρ*_α_, respectively.

A region where ∇^2^*s* < 0, *i.e.* LS_S_ > 0 increases the α component of the total electron density, *i.e.* the α-spin polarization, at a given rp **r**. Hereinafter, such capability will be termed as one leading to an ‘α effect’, the opposite behaviour, when ∇^2^*s* > 0, being instead named as one producing a ‘β effect’. Therefore, the ability of a point **r′** to act as a SDD source or sink for its neighbourhoods depends on the relative magnitude of ∇^2^*ρ*_α_ and ∇^2^*ρ*_β_ at that point (eqn (5)). For example, from Table 1 it can be seen that points 1–3 are all expected to provide a β effect, no matter the sign of the SDD at those points, as both the Laplacian components are negative and |∇^2^*ρ*_β_| > |∇^2^*ρ*_α_|.

At the lone pair nbcc (3 in Fig. 2), the spin density is small (from 0.003 to 0.022 a.u., according to the computational level) and the β-EDD almost twice more concentrated than the α one, as a reaction to the close large α-concentration due to the unpaired α-electrons. This implies that the region of the lone pair will produce, on average, a significant β effect, even though spin polarization is positive (but low) at its nbcc. On the contrary, at the two symmetric (3,+1) *L*(**r**) points (4 in Fig. 2) the spin density is by one to two order of magnitude larger, *ρ*_β_ depleted (∇^2^*ρ*_β_ > 0), and *ρ*_α_ highly concentrated (∇^2^*ρ*_α_ < 0). This results in ∇^2^*s*(**r**) ≪ 0, leading to large and positive regions of LS_S_ (α effect) around these points. In summary, even though both the regions dominated by the lone pair and the unpaired electrons are characterized by positive spin densities, they give rise to competing β and α effects, which the LS_S_ descriptor is able to unravel.

Each property listed in Table 1 may be then decomposed into a *magnetic* contribution arising from the two unpaired α-electron orbitals (hereinafter “magnetic orbitals”) and into a *reaction* or *relaxation* contribution due to the remaining orbitals. Magnetic orbitals, having B_1_ and A_1_ symmetry, are easily sorted out^24^ by diagonalizing the first order density matrix and by taking those natural orbitals with occupation *n* equal to or marginally different from one. For ^3^B_1_ H_2_O, the largest deviation is 0.0003 for one magnetic orbital of the CASSCF wavefunction, while *n* is exactly one for the ROHF wavefunction, which has, by definition, β-density and relaxation contribution both set to zero everywhere. A 3D plot of the two magnetic orbitals densities, as well of the total spin density, is reported in the ESI (Fig. S1–S3†) for the CASSCF(8,8) wavefunction. Note that for these orbitals, *ρ* ≡ *s*, ∇^2^*ρ* ≡ ∇^2^*s*, *ρ*_α_ ≡ *s*, ∇^2^*ρ*_α_ ≡ ∇^2^*s* while *ρ*_β_ and ∇^2^*ρ*_β_ are both null, so that only *s* and ∇^2^*s* values need to be reported (Table 1, values in parentheses).^25^ Note, also, that *s* and ∇^2^*s* contributions due to the remaining orbitals, as obtained by subtracting those of the magnetic orbitals from the total *s* and ∇^2^*s* values, may differ from zero at a given point, despite both contributions being null when integrated over the whole space. We observe (Table 1) that the magnetic orbitals dominate (i) the large spin density and its largely negative Laplacian at the two symmetric (3,+1) *L*(**r**) points 4, and (ii) the spin density depletion (∇^2^*s* > 0) at the in-plane non-bonded maximum 3 associated to the lone pair. Conversely, for the CASSCF and UHF models, the remaining orbitals overreact to the small positive *s*(**r**) contribution due to the magnetic orbitals at bcp 1. For the ROHF wavefunction, this reaction mechanism is unattainable and, differently from the CASSCF and UHF models, *s* remains positive at this CP. At the bcc 2, spin contributions from the two set of orbitals are equal in sign and definitely larger for the magnetic orbitals value. Nevertheless, the SDD of the magnetic orbitals is largely depleted (∇^2^*s* = 1.2 a.u.) while the SDD of the remaining orbitals is moderately concentrated (∇^2^*s* = –0.3 a.u. and –0.1 a.u. for the CASSCF and the UHF wavefunctions, respectively). This leads to a global dilution of the spin density in 2.

Upon introduction of static and (albeit limited) dynamic electron correlation at the CASSCF(8,8) level, one may generally observe (Table 1, Fig. 1 and 2) a similar qualitative picture relative to that at the UHF spin-contamination annihilated level. Such an agreement is even almost quantitative for the magnetic contributions (Table 1). A notable exception is found, however, for the spin density at the in-plane non-bonded maximum 3 associated to the lone pair. Electron correlation effects raise by more than five time its positive value, with such an increase being only due to the reaction or *relaxation* contribution (Table 1). Such an effect is also clearly visible in Fig. 2, where the small region of negative spin density of the UHF model lying close to the non-bonded maximum, disappears in the corresponding CASSCF plot. The effect (not shown) is even more evident if the UHF model spin contamination is not annihilated.

The ∇^2^*ρ*(**r**) and ∇^2^*s*(**r**) functions have noticeably different portraits. In water, ∇^2^*ρ*(**r**) implies relatively contracted valence shell charge concentration (VSCC) zones, mainly localized around nuclei and along covalent bonds, while the ∇^2^*s* < 0 regions are definitely more extended and possibly disjointed (Fig. 1 and 2). Furthermore, a given region of space may be diluted for *ρ*(**r**) and concentrated for *s*(**r**) or *vice versa*. Maps of *s*(**r**) and ∇^2^*s*(**r**) obtained from the magnetic and remaining natural orbitals and relative to the planes shown in Fig. 1 and 2, are reported in the ESI (S4–S7†).

Table 2 summarizes the relationship between the local relative magnitudes of the α- and β-density Laplacian distributions and the effect they determine at a given rp **r**. If *ρ*_α_ or *ρ*_β_ is locally concentrated, while the other distribution is locally depleted, the, respectively, overall α or β effect will be necessarily dominated by the concentrated distribution, no matter the relative magnitudes of the *ρ*_α_ or *ρ*_β_ Laplacians. In contrast, if both *ρ*_α_ and *ρ*_β_ are locally concentrated (depleted), the sign of LS_S_(**r**,**r′**) will be positive or negative (negative or positive) depending on whether it is the α or the β distribution that is more concentrated (depleted). Note that having both distributions concentrated or depleted does not ensure a positive (α) or a negative (β) effect. What matters, in both cases, is the relative concentration or dilution of the two distributions.

**Table 2:** How the signs and relative magnitudes of ∇^2^*ρ*_α_ and ∇^2^*ρ*_β_ at **r′** produce an α or β effect on the spin density *s* at the rp **r**

Sign[∇^2^*ρ*_α_(**r′**)]	Sign[∇^2^*ρ*_β_(**r′**)]	Relative magnitudes	∇^2^*s*(**r**)	LS_S_(**r**,**r′**)	Effect on *s*(**r**)
>0	>0	∇^2^*ρ*_α_ > ∇^2^*ρ*_β_	>0	<0	β
		∇^2^*ρ*_α_ < ∇^2^*ρ*_β_	<0	>0	α
>0	<0	Any	>0	<0	β
<0	>0	Any	<0	>0	α
<0	<0	|∇^2^*ρ*_α_| > |∇^2^*ρ*_β_|	<0	>0	α
		|∇^2^*ρ*_α_| < |∇^2^*ρ*_β_|	>0	<0	β

## Atomic spin and electron density source function

### A test case: ^3^B_1_ water

After having discussed what establishes whether a (infinitesimal) region of space is acting as a source or sink for the SDD, we now investigate how the spin density at the chosen rps of ^3^B_1_ H_2_O is reconstructed in terms of its SF_s_ atomic contributions (eqn (4)). Moreover, we explore whether any chemical insight may be retrieved by decomposing the spin density in terms of such *non-local* effects.

For our adopted model wavefunctions, Bader's atomic spin populations in ^3^B_1_ H_2_O amount to 0.29/0.31 (H) and 1.42/1.39 (O), indicating that ≈2/3 of the unpaired electrons are localized in *Ω*(O). The ∇^2^*s*(**r**) distribution, at the same time, integrates to 0.02 a.u. in the H basin and to –0.04 a.u. in the O basin, for all models. These values can be interpreted as the influence exerted by each atom at great distance, when the 1/|**r** – **r′**| Green's factor (eqn (5)) is small enough to be safely taken out from the integral as a constant. Therefore, H atoms in ^3^B_1_ H_2_O tend to exploit a β effect, while O is expected to act as an α source at a great distance. However, the actual sign of the integral SF_S_ descriptor (eqn (4)) will depend on the choice of the rp point which determines through the Green's factor the relative weight of the local cause ∇^2^*s*(**r′**) in the various regions of the integrated atom.^26^

**Fig. 3:**
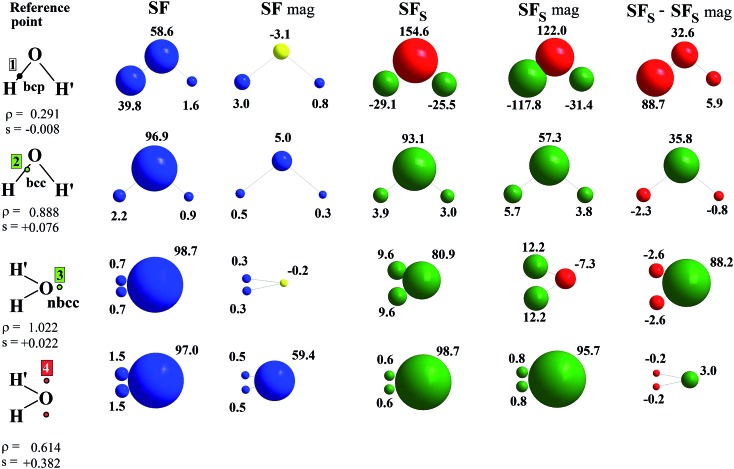
SF and SF_S_ percentage contributions at some reference points (rps) for ^3^B_1_ H_2_O at the CASSCF(8,8) level. The separate contributions to SF_S_ due to the magnetic (SF_S_ mag) and the remaining (SF_S_ – SF_S_ mag) natural orbitals are also shown (for SF only those due to magnetic orbitals, denoted as SF mag). Each atom is displayed as a sphere, whose volume is proportional to the source percentage contribution to *ρ*(**r**) or *s*(**r**) values at the rp (first column). Colour codes: blue (yellow) atoms act as positive (negative) sources for *ρ* at rps; green (red) atoms act as positive (negative) sources for *s* at rp, hence yielding an α(β) effect at rp (the sign of percentage source is instead positive or negative whether the atomic source concurs with or opposes *s* at rp).

Fig. 3 shows the relative percentage contributions of SF and SF_S_ from individual atomic basins of the ^3^B_1_ H_2_O molecule at the previously considered rps (see Fig. 1 and 2 and Table 1) and for the CASSCF(8,8) model. Results for spin-contamination annihilated UHF and for the ROHF wavefunctions are reported in the ESI (Fig. S8 and S9†).

The corresponding absolute values are listed in Table 3 for all investigated models, while in section 2 of the ESI (ESI_2)† detailed information on the numerical accuracy of the spin density reconstruction in terms of SF_S_ contributions is reported.

**Table 3:** SF and SF_s_ values (atomic units) in ^3^B_1_ H_2_O as a function of the computational level and with contributions due to magnetic natural orbitals given in parentheses^*a*^ ^*b*^

Point	H		O		H′	
	SF	SF_s_	SF	SF_s_	SF	SF_s_
**CASSCF(8,8)//UHF/6-311++G(2d,2p)**						
1	0.1155	0.0022 (0.0087)	0.1704	–0.0115 (–0.0091)	0.0046	0.0019 (0.0023)
2	0.0192	0.0030 (0.0044)	0.8585	0.0713 (0.0439)	0.0080	0.0023 (0.0029)
3	0.0068	0.0021 (0.0027)	1.0088	0.0177 (–0.0016)	0.0068	0.0021 (0.0027)
4	0.0091	0.0024 (0.0031)	0.5953	0.3761 (0.3644)	0.0091	0.0024 (0.0031)

**UHF/6-311++G(2d,2p) spin-contamination annihilated wavefunction**						
1	0.1109	0.0063 (0.0106)	0.1725	–0.0137 (–0.0102)	0.0041	0.0024 (0.0026)
2	0.0170	0.0042 (0.0049)	0.8622	0.0561 (0.0433)	0.0073	0.0030 (0.0033)
3	0.0061	0.0027 (0.0030)	1.0178	–0.0004 (–0.0022)	0.0061	0.0027 (0.0030)
4	0.0082	0.0032 (0.0035)	0.5937	0.3740 (0.3594)	0.0082	0.0032 (0.0035)

**ROHF//UHF(6-311++G(2d,2p)** ^*c*^						
1	0.1104	0.0108	0.1724	–0.0102	0.0041	0.0026
2	0.0168	0.0049	0.8643	0.0404	0.0072	0.0033
3	0.0061	0.0030	1.0188	–0.0027	0.0061	0.0030
4	0.0081	0.0035	0.5901	0.3554	0.0081	0.0035

^*a*^Values reported in this table for SF and SF_s_ yield the percentage source contributions at the 1–4 reference points shown in Fig. 3 (CASSCF) and in Fig. S8 (UHF), S9 (ROHF) of the ESI.

^*b*^The source contributions of magnetic natural orbitals to SF(*Ω*) equal by definition those to SF_s_(*Ω*) and are thus not reported in the table, while their related % source contributions clearly differ (see Fig. 3 and S8 and S9).

^*c*^For the ROHF wavefunction, *s* ≡ *s*_mag_ and thus SF_s_ ≡ SF_S_ mag.

When the O–H bcp (1) is considered, the SF picture is coherent with a classical covalent polar bond scenario, with the more electronegative O atom providing ≈60% of the electron density at the rp. The remaining ≈40% is due to the bonded hydrogen H, while its symmetry-related H′ atom has an almost negligible influence. On the other hand, the information supplied by the SF_S_ descriptor is completely different. As *s*(**r**) < 0 at the O–H bcp (Table 1) for the CASSCF and UHF models enabling spin relaxation, a positive percentage contribution means a β effect in this context, *i.e.* a negative contribution to *s*(**r**). This is just the case for the O basin, while both H atoms counteract its influence through an α effect, as could be foreseen by the extended zone of negative ∇^2^*s*(**r**) in their basins (Fig. 1).^27^ This picture reflects and quantifies a spin polarization mechanism, where the full pairing of covalent O–H bonds in the X ^1^A_1_ water ground state is perturbed by the presence of unpaired electrons in the triplet excited state. Moreover, at variance with SF, the symmetry-related H′ atom provides the *ρ*(**r**) bcp with a quite large SF_S_ contribution, very similar to that from H at the CASSCF level. This suggests that the spin polarization in the molecular plane takes place both through bond and through space mechanisms. In any case, both mechanisms imply that the strong β effect at the bcp due to oxygen is partly (CASSCF) or largely (UHF) counteracted by hydrogen atoms. Note also that all three considered computational models yield qualitatively similar SF_s_(H′) contributions, while the UHF and in particular the ROHF model largely overestimate the counteracting α-effect of the H atom associated to the bcp (respectively about three and five times larger than for the CASSCF model). Further insight is provided by examining the separate contributions to SF_S_ due to the magnetic (SF_S_ mag) and the remaining other orbitals (SF_S_ – SF_S_ mag) (Fig. 3, S8 and S9 and Table 3). As a premise, we note that other orbitals contribute to ≈99% and ≈127% of the CASSCF bcp electron and electron spin densities, respectively, but a more detailed analysis is revealing. Both the magnetic and the remaining orbitals concur to the strong β effect at the bcp due to the O atom (+122.0% and +32.6%, respectively), while the similar counteracting α effect played by the two H atoms has a clearly distinct origin. For the bonded H atom the quite large α effect due to magnetic orbitals (SF_s_ mag, –117.8%) is to a great extent (+88.7%) compensated for by the β effect prompted by the remaining (bonding) orbitals. Conversely, for H′, the latter orbitals have, as expected, an almost negligible influence (+5.9%) but the effect of the magnetic ones, though lowered to one fourth of the strength they have for H by the increased distance from the bcp, still remains significant (SF_s_ mag, –31.4%).^28^

Due to the close proximity to the O atom, both *ρ*(**r**) and *s*(**r**) are largely dominated by this atom at the bcc 2. Herein, *s*(**r**) is positive, about one order of magnitude larger than at bcp (Table 1) and similarly determined by the magnetic and remaining orbitals, with the former yielding α contributions for both O and H atoms. At the two symmetric points 4, *s*(**r**) is two order of magnitude larger than at bcp and, similarly to *ρ*(**r**), almost all determined by the O atom (Fig. 3, S8 and S9†). At variance with bcc 2, however, the magnetic orbitals here largely dominate (CASSCF, 97.3%) the *s*(**r**) value. In turn, over 93% of their contribution at CASSCF level comes from the magnetic orbital B_1_ since points 4 are representative of the α-spin density described by the O[p_*x*_] functions. Also the SF contributions to *ρ*(**r**) enable one to distinguish the different natures of the two points: at bcc 2 the contribution from magnetic orbitals is marginal (CASSCF, 5.8%), while at points 4 it is about ten times larger (60.4%), though clearly not as dominant as it is for *s*(**r**). At the nbcc 3, representative of the O lone pair, *s*(**r**) is positive and, as noted earlier, with magnitude largely dependent on the wavefunction model. While the electron density value is overwhelmingly dominated by the O atom for all models, *s*(**r**) is at the UHF and ROHF levels overdetermined (UHF: 108%; ROHF: 183.1%) by the hydrogen atoms, although the nbcc lies on the opposite side of these atoms. In contrast, upon introduction of static and dynamic correlation at the CASSCF(8,8) level, one recovers a much less unanticipated result, as H atoms and the O atom contribute, respectively, to 19% and 81% of the *s*(**r**) value. Such different behaviour finds an easy explanation in terms of separate orbital contributions. The large α-effect from the H atoms results in the UHF model from a dominant α-contribution due to the magnetic orbitals, slightly opposed by the β-effect due to the remaining orbitals, while for the O atom these orbital effects are reversed and the β-effect of the magnetic orbitals slightly prevails. Out of the two, it is only the A_1_ totally symmetric magnetic orbital which plays the role in such mechanisms. It allows the H atoms to exert a direct influence on the positive spin density at the nbcc, while causing the O atom to partly oppose such an influence. In the case of the ROHF wavefunction, the SF_S_ mag (≡SF_S_ for ROHF) contributions from O and in particular from H atoms are very much alike in magnitude to those of the UHF model (Table 3), but owing to the lack of the spin relaxation mechanism, the dominance of the H atoms α-effect is even largely enhanced for ROHF (compare Fig. S8 and S9†). The effect of including a larger amount of electron correlation (CASSCF model) is to enhance by one order of magnitude, from 0.0018 (UHF) to 0.0193 a.u., the contribution to SF_S_(O) from non-magnetic orbitals, while that from magnetic orbitals is very similar in the two models, both for O and H atoms. As a consequence the percentage SF_S_ sources for the CASSCF and the UHF (or ROHF) models at nbcc 3 look very different among each other (Fig. 3 and S8 and S9†).

It is worth noting that SF_S_ contributions, and in particular their magnetic and non-magnetic components, neatly distinguish the different chemical natures of points representative of unpaired-electron or lone-pair electrons charge concentrations, while the corresponding SF values do not (Fig. 3 and S8 and S9†).

## General remarks and conclusions

In this work, the Spin Density Source Function (SDSF) was introduced as a new tool to highlight how spin information propagates from paramagnetic to non-magnetic centers and how these latter may in turn influence the spin density distribution of the paramagnetic center. SDSF recovers the spin density at a point in terms of separate atoms or group of atoms contributions. The way the paramagnetic center spin-polarizes the non-magnetic centers or the extent that these latter back-determine the spin distribution of the former strongly depends on the chosen points of analysis (reference points). This occurs because of the large anisotropy of the spin and, even more so, of the spin Laplacian distributions within atomic basins. Indeed, it may result that the spin density at a point is almost fully determined by the atomic basin to which the point belongs, but the opposite may also occur, and even in the case of regions within the basin of the paramagnetic center. This is namely the case of the charge concentration maximum associated to the O atom lone pair in the water triplet, when only the limited electron correlation enabled by the UHF model is included. The very low positive spin density value found at this point, lying only 0.33 Å away from the oxygen and on the opposite side with respect to the hydrogen atoms, is even overdetermined (SF_s_(H + H′)% = 108) by the two distant H atoms. Chemical interpretation of SDSF atomic contributions is enhanced by decomposing them in a *magnetic* term due to the magnetic natural orbital(s) density and in a *reaction* or *relaxation* term due to the remaining natural orbitals density. The reasons leading, in the water triplet, to dominant oxygen atom contributions and to dominant hydrogen atoms contribution for the UHF spin density at points respectively associated to unpaired and lone pair electrons, have been rationalized this way. As it was the re-established dominance of the oxygen contribution also to the spin density at the lone pair position, when electron correlation at the CASSCF level is included. This latter leaves almost unaffected the O and H atoms magnetic contributions to the spin density at such position, while it selectively increases the O relaxation contribution by one order of magnitude relative to the UHF model. We have also shown that the magnetic term does not necessarily determine a positive spin density at any reference point, but may instead produce what we called a β-effect, that is a decrease of the local spin density. Furthermore, the relaxation term may either concur or counteract the magnetic term in determining the spin density at a given point, regardless of its link to an orbital density integrating to a null spin population over the whole space. Actually, the SDSF atomic contributions and their magnetic and reaction components, are all obtained through the atomic integration of the corresponding local source functions, which are given in terms of the related spin density Laplacians. These latter, regardless of the sign of s(**r**), may be locally positive or negative depending on the local concentration/dilution of the corresponding α- and β-densities.

Dissecting SDSF atomic contributions into a magnetic and a reaction component enhances interpretability although it might be seen as arbitrary. Use of natural orbitals from an approximate density matrix minimizes such risk. The number of unpaired electrons *n* and their spatial localization (“magnetic orbitals”) can in principle be determined from the diagonalization of the exact density matrix of the N-electron ground-state wave function,^24^ by singling out those *n* natural orbitals having occupation numbers very close to one. These are hypothetical magnetic orbitals, because it is impossible to obtain an exact wavefunction for any non-trivial system. Using wavefunction models at increasing level of complexity should, however, yield magnetic orbitals closer and closer to the hypothetical ones. Our results are encouraging as they suggest that even a very simple wavefunction model like ROHF or UHF leads to magnetic orbitals which are very much alike to those of a clearly more complex model [CASSCF (8,8)], in terms of both their local properties at the critical points of the total electron density or electron density Laplacian and of their electron and electron spin densities source functions contributions. Increasing the wavefunction quality has instead a noticeable effect on the reaction or relaxation component. Source function analysis may thus be proposed as a useful tool leading to an atom by atom (and point by point) *quantitative insight* of the influence of the wavefunction model on such components.

One should also not ignore that singling out magnetic orbitals may become difficult when highly correlated cases, requiring more than one reference configuration and where natural occupancies will approach neither 2 nor 1, are afforded.^29^ Recently developed techniques to obtain one-electron functions from real space partitionings of the molecular space – the so called domain natural orbitals and multicenter natural adaptive orbitals – might likely serve the scope.^30^–^32^

Joint analyses of the spin and electron density source functions provide interesting insight, since the reconstruction of the spin density in terms of atomic source function contributions may be similar or largely differ from the one for the electron density. This is respectively the case of the points associated to the unpaired and lone pair electrons in the water triplet. Separate analysis of the magnetic and reaction or relaxation terms of the spin density source function contributions clearly elucidates why.

Being defined in term of an observable, the source function for the spin density is also potentially amenable to experimental determination, as already largely exploited for its electron density analogue.^15^–^19^,^23^ The future possibility of an unbiased direct comparison of *ab initio* and experimentally (PND + X-ray) derived results is of paramount importance in view, on the one hand, of the large sensitivity of spin densities to the adopted theoretical framework and, on the other hand, of the technical limitations and of the multipole modelling ambiguities associated to the experiment. Decomposition of the experimental SDSF atomic contributions into magnetic and reactions components could be still easily afforded by assigning their values through a partitioning function, given on a grid and defined through the relative weights of the corresponding components from theory.

## Conflict of interest

The authors declare no competing financial interests.

## Supplementary Material

Supplementary information
