# Supplementary material for: Insights on spin polarization through the spin density source function
Source: Chem Sci. 2015 Apr 14;6(7):3845–52. doi: 10.1039/c4sc03988b (PMC5707457; doi:10.1039/c4sc03988b)
Supplement: Supplementary file 1 [file SC-006-C4SC03988B-s001.pdf]

Electronic Supplementary Material (ESI) for Chemical Science.
This journal is © The Royal Society of Chemistry 2015

Insights on Spin Polarization through the Spin Density Source Function

Carlo Gatti[*,+,∀], *Ahmed M. Orlando*[∀,#] *and Leonardo Lo Presti*[+,∀,#]

[+]CNR-ISTM, Istituto di Scienze e Tecnologie Molecolari, Via Golgi 19, 20133 Milano, Italy

[∀] Center for Materials Crystallography, Aarhus University, Langelandsgade 140, 8000 Aarhus, Denmark

[#] Dipartimento di Chimica, Università degli Studi di Milano, Via Golgi 19, 20133 Milano, Italy

AUTHOR INFORMATION

Corresponding Author

* Carlo Gatti, CNR-ISTM, Via Golgi 19, 20133 Milano (Italy). E-mail: c.gatti@istm.cnr.it

Electronic Supplementary Information (ESI)

ESI_1. Computational Methods and new software codes

All the quantum mechanical simulations for $^3B_1$ H_2O were performed *in vacuo* by means of the Gaussian09 program package.[1] The CASSCF(8,8), UHF (Unrestricted Hartee Fock) , ROHF (Restricted Open Hartree Fock) levels of theory were all employed with a 6–311++G(2d,2p) basis set. Computations on both spin-contamination annihilated and spin contaminated UHF wavefunctions were performed. Spin contamination by states of higher multiplicity than the triplet state was minor , $<S^2>= 2.0069$, and almost zero after the annihilation procedure, $<S^2>= 2.000014$. Starting guess for the CASSCF computation was taken from the UHF spin contamination annihilated Natural Orbitals. The Slater determinant expansion of the CASSCF(8,8) wavefunction includes 3136 configurations of the correct symmetry and spin multiplicity. Natural orbitals were obtained through the pop= no option and magnetic orbitals very easily singled out based on their occupation numbers, in all cases.[2] For ROHF wavefunction they have by definition occupation numbers equal to one, for the other wavefunctions they were either one or marginally different from one (highest deviation from one being 0.0003). Note that Spin densities were instead calculated from the naturals orbitals obtained from separate diagonalizations of the α- and β-density matrices (pop=noab option). In the case of CASSCF method, Gaussian09 is apparently unable to calculate and save spin density information. However, by activating the IOP(5/72=1) option and introducing a “1 1” at the bottom of input file before the name selected for the .wfn file, spin density information is passed to L6XX (XX=01,…etc) links. Slater determinants (SlaterDet option in CASSCF9) should be used in this case in the CASSCF calculation. Operating this way, one eventually recovers a correct α-density through the pop=noa option (but, surprisingly, not the correct spin density through pop=noab, nor the correct β-density through pop=nob). From the total density and the α-density one then easily obtains the electron spin density and electron spin density Laplacian by difference: $s(\mathbf{r}) = 2\rho_\alpha(\mathbf{r}) - \rho(\mathbf{r})$; $\nabla^2 s(\mathbf{r}) = 2\nabla^2\rho_\alpha(\mathbf{r}) - \nabla^2\rho(\mathbf{r})$.

As a further hint to the reader, we report that the only way to save the UHF spin contamination annihilated solution on the .wfn file is to use the “pop” option involving natural orbitals (e.g.

pop=no, pop=noab), otherwise the spin-contaminated wavefunction is saved even if the proper option for UHF spin annihilation [IOP(5/14=2)], is activated. The .wfn file serves as a wavefunction input for the AIMPAC program package (see below).

Topological analysis of the various scalar fields was performed through a modified version of the AIMPAC program package.[3] The Fortran 77 routines for computing the local and integrated Source Function for the spin density were implemented by one of us (C. G.) to yield a number of source codes enabling the complete study of such functions. Namely:

1. **spinsf**: A heavily modified version of the original PROMEGA/PROAIMV code, enabling one to evaluate atomic Source Function contributions from electron and spin densities;

2. **extremespin**: A modified version of the original EXTREME code, enabling the evaluation of the spin density and of the Laplacian of the spin density, along with their separate α and β contributions;

3. **plotden2013**: A code to evaluate contour maps in hpgl format for several scalar and vector fields, including the spin density, its Laplacian and the local source functions for the electron and electron spin densities, given a specific reference point.

4. **profilbpsource2013**: A code for evaluating several scalar functions in specific points or along lines, including bond paths. More in detail, the program can handle spin density and its Laplacian, local source for the electron and electron spin densities, individual ρ_α , ρ_β , $\nabla^2\rho_\alpha$, $\nabla^2\rho_\beta$ contributions, bond ellipticity, local kinetic energy source, local potential energy source, etc..

Details on the accuracy of atomic integrations required to adequately reconstruct the spin density distribution in terms of the Source Function atomic contributions are given in ESI_2

The code Diamond v3.21, © 1997–2012 Crystal Impact GbR, Bonn, Germany was employed to draw all the ball-and-stick pictures, while the VESTA[4] program was used to draw isosurface plots.

Table S1. SF and SF_s absolute values (in a.u.) in $^3B_1$ H_2O, as evaluated for (i) the whole set of molecular orbitals (MO) and (ii) the two A_1 and B_1 symmetry magnetic orbitals. Values reported in this Table correspond to the relative percentage contributions at the 1–4 reference points shown in Figure 3 (see the main text for details).

		ALL MO's		Magnetic orbitals		ALL MO's – Mag. Orb.	
rp	ATOM	ρ(**r**)	s(**r**)	ρ(**r**)	s(**r**)	ρ(**r**)	s(**r**)
1	H2	0.111	0.002	0.012	0.012	0.098	-0.010
	O1	0.171	-0.017	-0.011	-0.011	0.182	-0.006
	H3	0.004	0.002	0.003	0.003	0.001	-0.001
2	H2	0.017	0.004	0.005	0.005	0.012	-0.002
	O1	0.862	0.069	0.033	0.033	0.829	0.036
	H3	0.007	0.003	0.004	0.004	0.004	-0.001
3	H2	0.006	0.003	0.003	0.003	0.003	-0.001
	O1	1.018	0.001	-0.003	-0.003	1.021	0.004
	H3	0.006	0.003	0.003	0.003	0.003	-0.001
4	H2	0.008	0.003	0.004	0.004	0.004	-0.001
	O1	0.594	0.389	0.355	0.355	0.240	0.034
	H3	0.008	0.003	0.004	0.004	0.004	-0.001

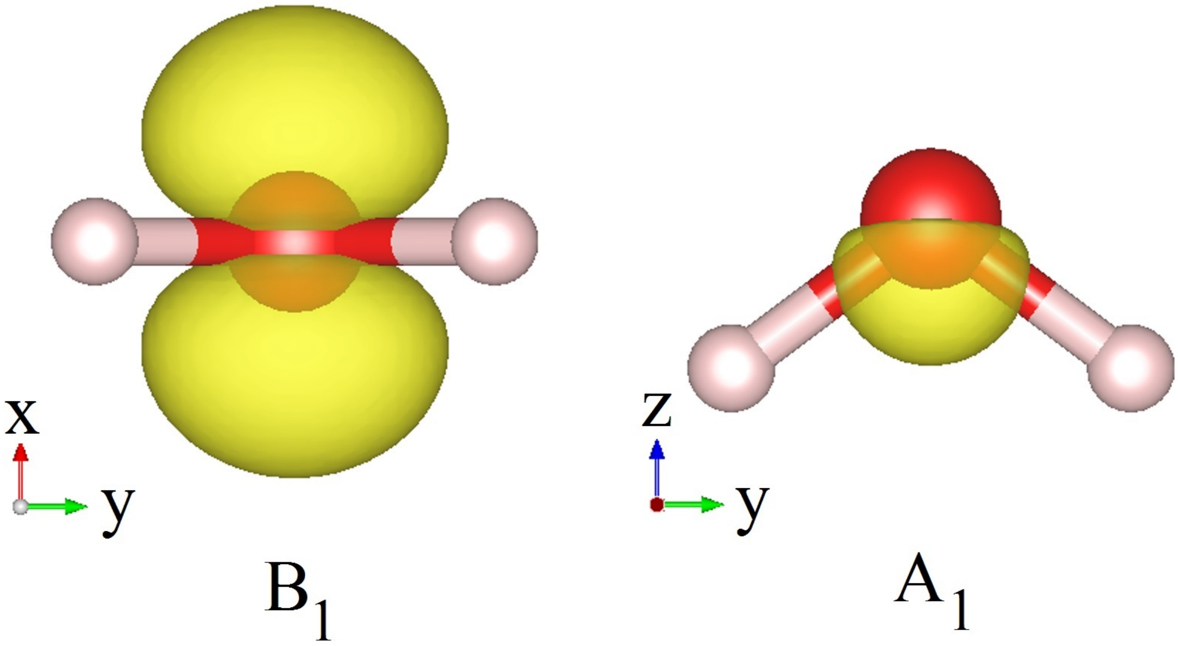

Figure S1. 3D spin density plots in the (*x,y*) and (*z,y*) plane, as evaluated just for the B_1 and A_1 symmetry magnetic natural orbitals at the CASSCF(8,8) level. An isosurface value of 0.015 a.u. was selected, with maxima of spin density equal to 0.596 a.u. for B_1 symmetry orbital and 0.250 a.u. for A_1 symmetry orbital.

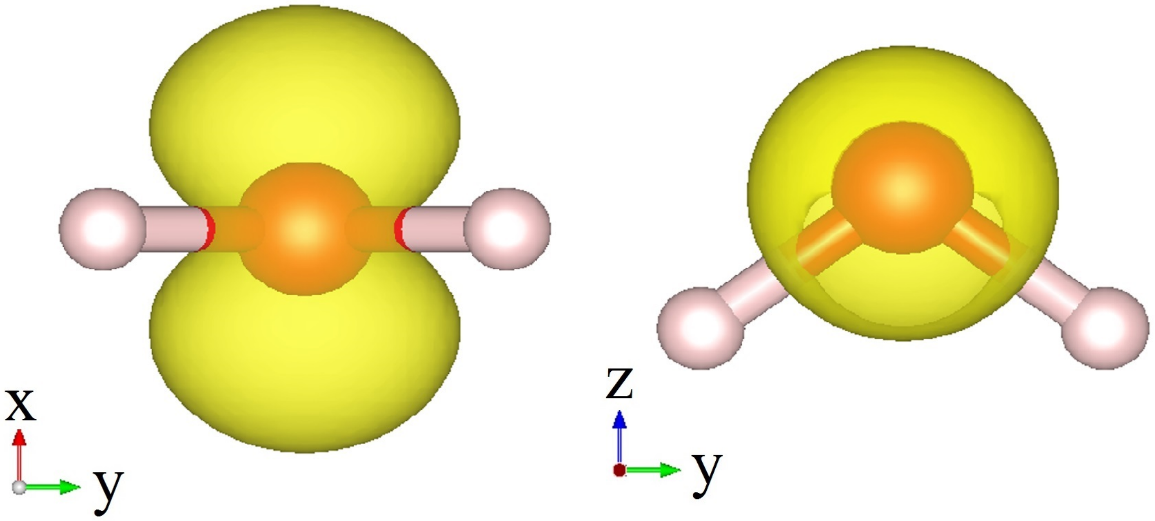

Figure S2. Same as Figure S1 above, but summing up the spin density contributions of the B_1 and A_1 symmetry magnetic natural orbitals. Maxima of spin density fall at 0.603 a.u.

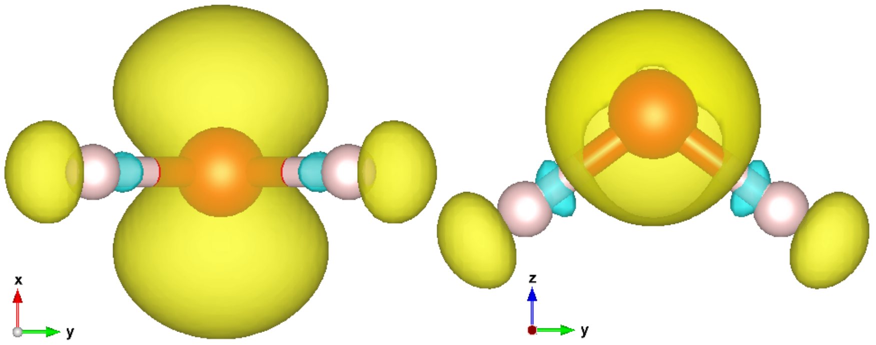

Figure S3. As Figures S1 and S2 above but plotting the total spin density. The maxima and minima of spin density fall at 0.618 a.u and -0.008 a.u. respectively.

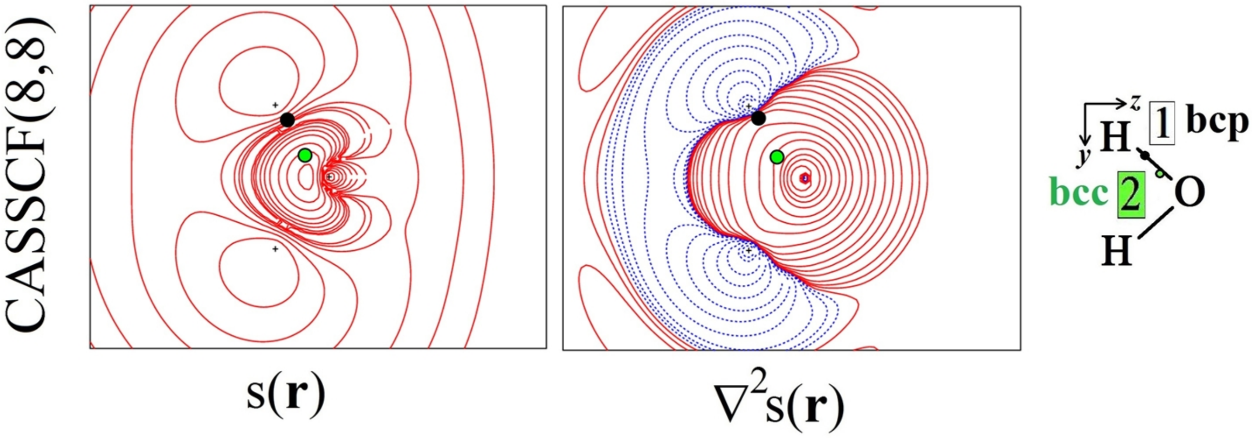

Figure S4. The spin density s(**r**), its Laplacian (∇^2s(**r**)) in the (y,z) plane for $^3B_1$ H_2O due just to the A_1 and B_1 symmetry magnetic natural orbitals. Atomic units (a.u.) are used throughout. Contour maps are drawn at interval of $\pm(2,4,8)\cdot 10^n$, $-4 \le n \le 0$ (s, ∇^2s) and $-3 \le n \le 0$ ($\nabla^2\rho$). Dotted blue (full red) lines indicate negative (positive) values. The O–H bond critical point (bcp, 1) and the bonded charge concentration point (bcc, 2) are shown as black and green dots.

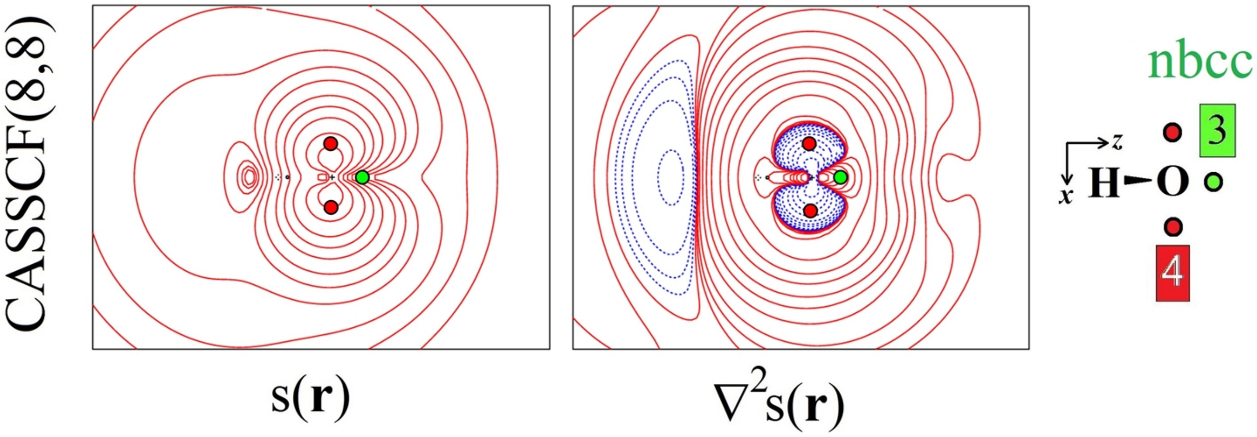

Figure S5. As Figure S4 above, in the (x,z) plane with the same contour levels. The non-bonded charge concentration (nbcc, 3) and the (3,+1) L(**r**) rcps (4) are shown as green and red dots.

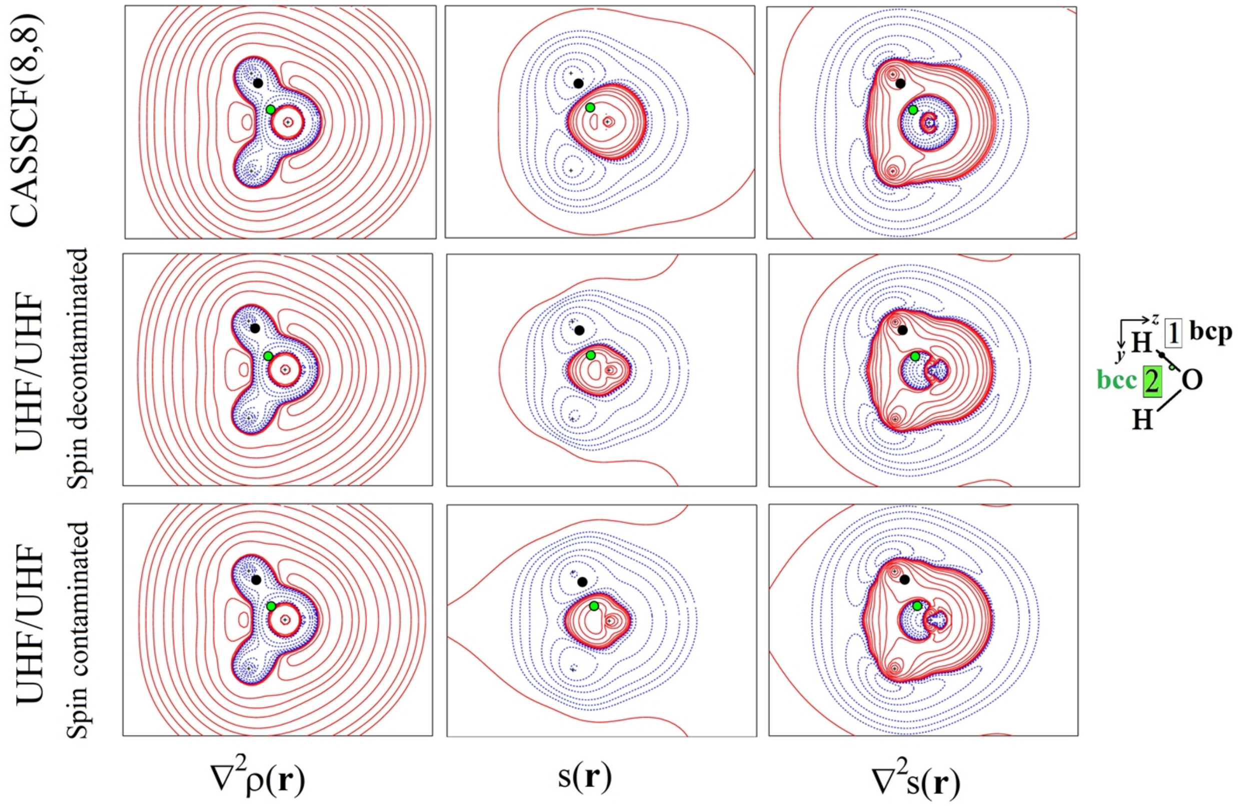

Figure S6. The spin density s(**r**), its Laplacian (∇²s(**r**)) and the electron density Laplacian, (∇²ρ(**r**)) in the (*y*,*z*) plane for $^3B_1$ H_2O due just to the non-magnetic natural orbitals for the CASSCF(8,8), the UHF/UHF spin-contamination annihilated and the UHF/UHF spin-contaminated models. Atomic units (a.u.) are used throughout. Contour maps are drawn at interval of $\pm(2,4,8)\cdot 10^n$, $-4 \le n \le 0$ (s, ∇²s) and $-3 \le n \le 0$ (∇²ρ). Dotted blue (full red) lines indicate negative (positive) values. The O–H bond critical point (bcp, 1) and the bonded charge concentration point (bcc, 2) are shown as black and green dots.

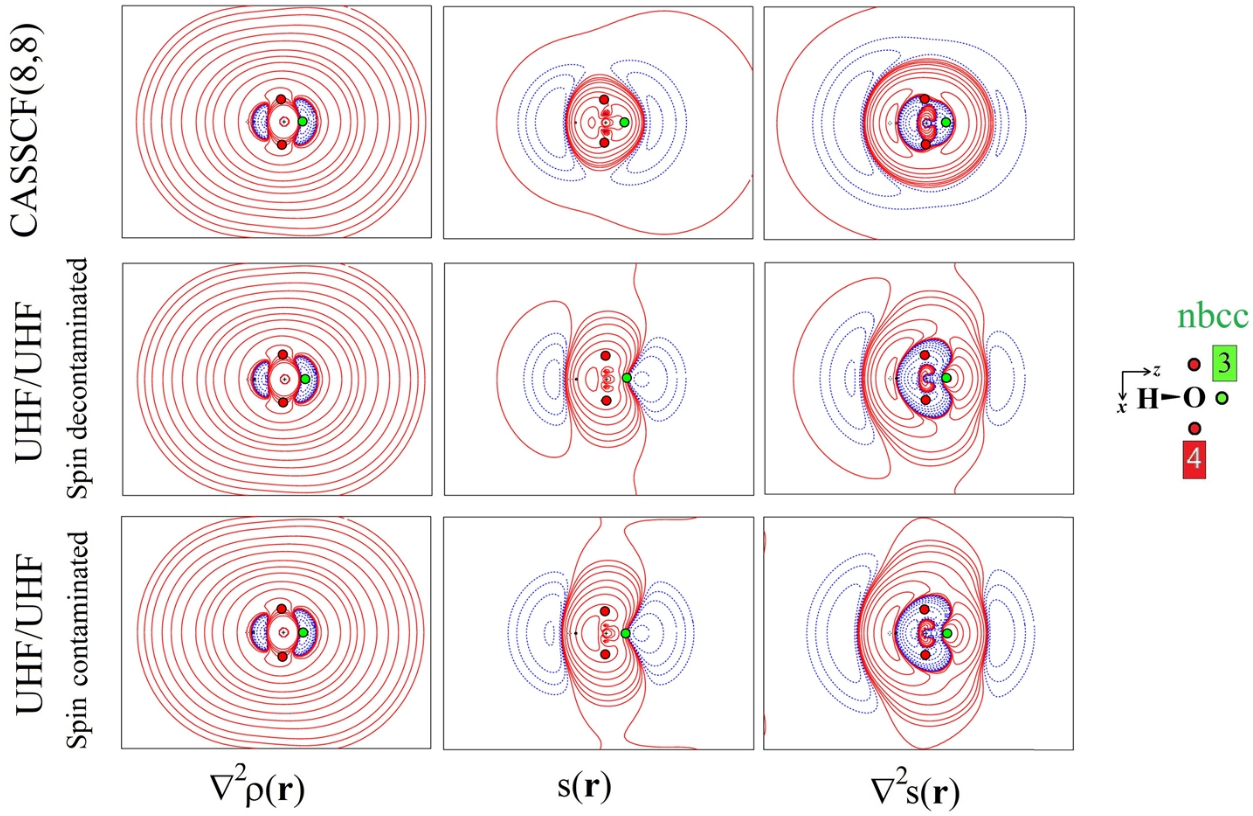

Figure S7. As Figure S6 above, in the (x,z) plane with the same contour levels. The non-bonded charge concentration (nbcc, 3) and the (3,+1) L(**r**) rcps (4) are shown as green and red dots.

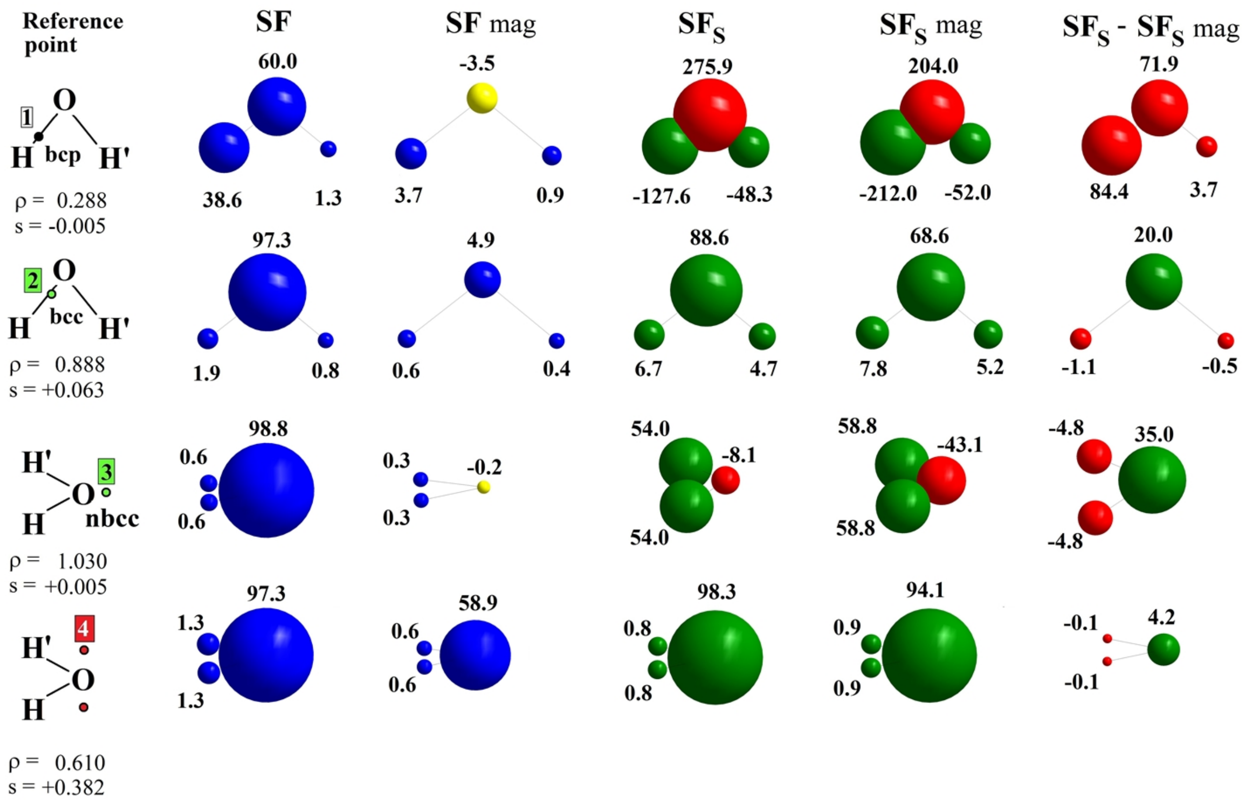

Figure S8. SF and SF$_S$ percentage contributions at some reference points (rps) for $^3B_1$ H_2O at the UHF/UHF spin contamination annihilated level. The separate contributions to SF$_S$ due to the magnetic (SF$_S$ mag) and the remaining (SF$_S$ - SF$_S$ mag) natural orbitals are also shown (for SF only those due to magnetic natural orbitals, denoted as SF mag). Each atom is displayed as a sphere, whose volume is proportional to the Source percentage contribution to ρ(**r**) or s(**r**) values at the rp (first column). Colour codes: blue (yellow) atoms act as positive (negative) sources for ρ at rps; green (red) atoms act as positive (negative) sources for s at rp, hence yielding a α (β) effect at rp (the sign of percentage sources is instead positive or negative whether the atomic source concurs or opposes to s at rp).

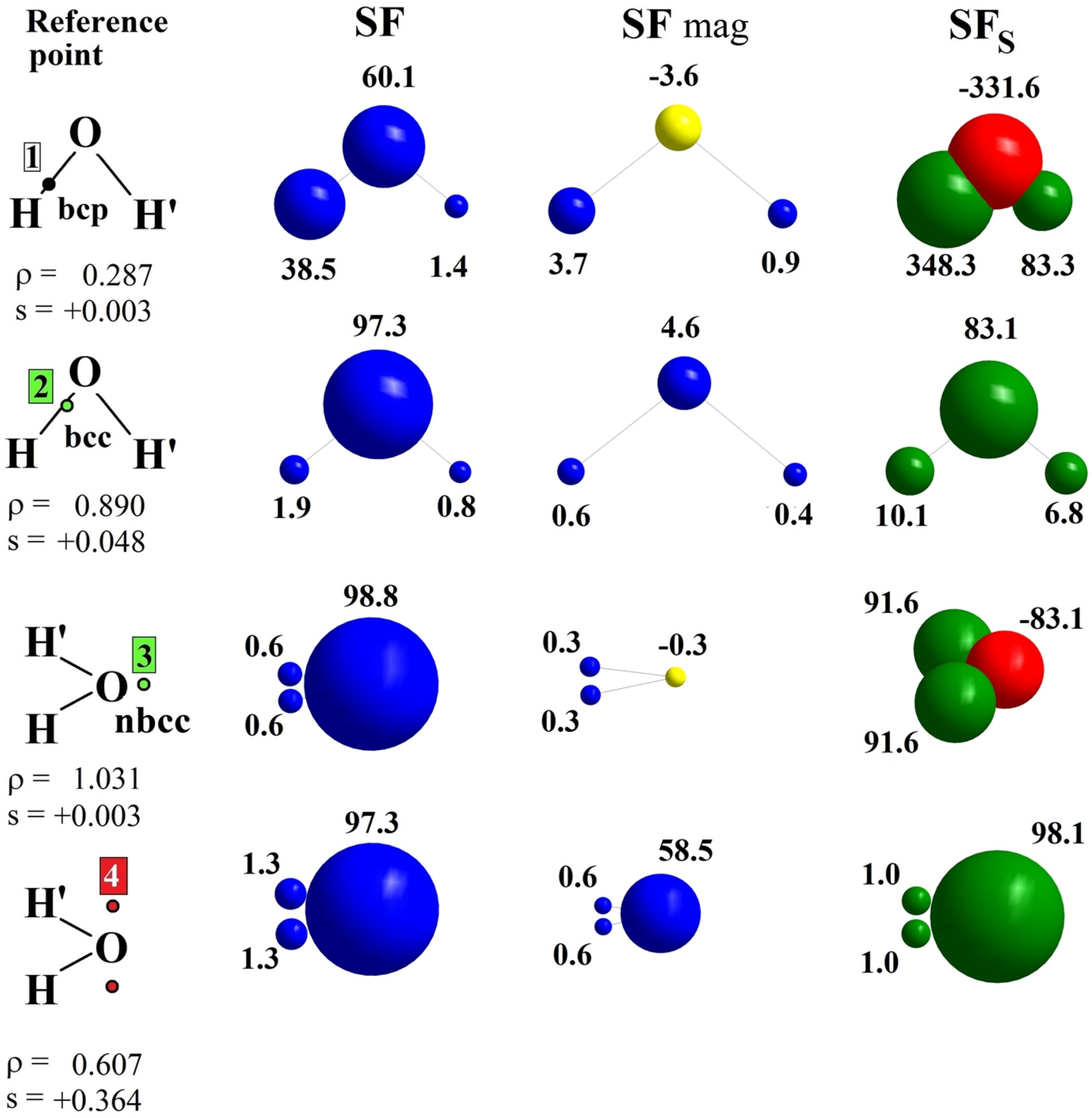

Figure S9. SF and SF_S percentage contributions at some reference points (rps) for $^3B_1$ H_2O at the ROHF//UHF spin contamination annihilated geometry level. The separate contributions to SF and SF_S due to the magnetic natural orbitals are also shown (SF mag and SF_S mag, respectively). Each atom is displayed as a sphere, whose volume is proportional to the Source percentage contribution to ρ(**r**) or s(**r**) values at the rp (first column). Colour codes: blue (yellow) atoms act as positive (negative) sources for ρ at rps; green (red) atoms act as positive (negative) sources for s at rp, hence yielding a α (β) effect at rp (the sign of percentage sources is instead positive or negative whether the atomic source concurs or opposes to s at rp).

ESI_2. Numerical accuracy of the spin density reconstruction.

As noted in the main body of the paper, for most representative points s(**r**) turns out to be relatively close to zero, which is not unexpected, given its very nature of a difference density (ρ_α - ρ_β). Indeed, values as low as 10^{-3} or 10^{-4} a.u. are common even near the nuclei and at the bcp's in the covalent bonding region. Moreover, sometimes the SF_S percentage contributions are very high in magnitude, even by far larger than 100%, and bear opposite signs. All these factors concur in foreseeing difficulties for an accurate s(**r**) reconstruction, although the plausibly dampened oscillations of ∇^2s(**r**), relative to $\nabla^2\rho$, should in part alleviate the problem.

We deemed, therefore, important: *i)* to assess the degree of numerical accuracy that is obtained through the s(**r**) Source Function reconstruction, and *ii)* to explore whether such an accuracy is reasonably uniform throughout the molecular space.

To this end, according also to a previous work of our group[5] we define an accuracy index *f*2 for the SDD reconstruction at the reference point **r**:

$$f2 = \frac{s(\mathbf{r}) - \sum_{\Omega} \mathrm{SF_S}(\mathbf{r},\Omega)}{s(\mathbf{r})} \cdot 100 \qquad (1)$$

Note that an analogue quantity *f*1 can be defined for the charge density by substituting in (1) s(**r**) with ρ(**r**), and SF_S with SF. Figure S9 shows the plots for *f*1 and *f*2 for $^3B_1$ water (UHF, spin-

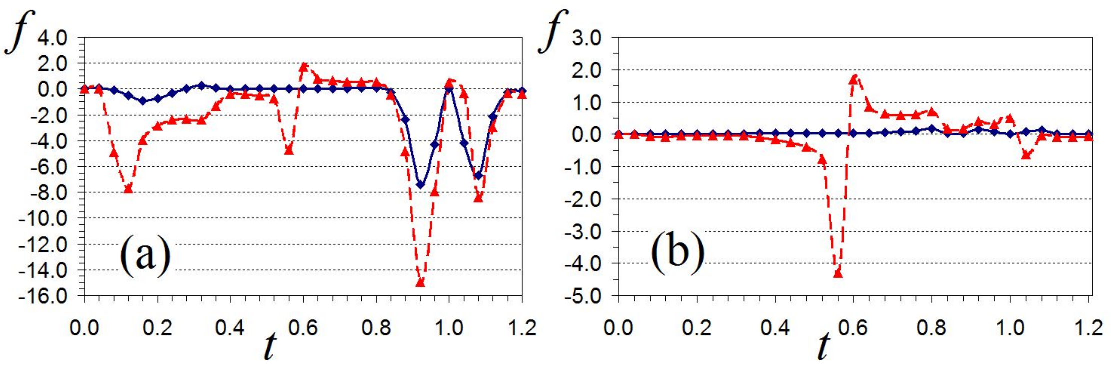

Figure S9. Accuracy indices (see text) for reconstructing $\rho(t)$ (rhombi, full blue line, *f*1) and $s(t)$ (triangles, dashed red line, *f*2) in $^3B_1$ water through the Source Function, *t* being the linear parametric distance from the O nucleus along the O–H bond (*t* = 0: O; *t* = 1: H). (a) Standard β-sphere integration; (b) Finer β-sphere integration.

contaminated model) along one symmetry-independent O–H bond so as to explore core, valence, and bonding regions and where the two panels refer to two different integration schemes (*see infra*). More in detail, we compare the accuracy retrieved for both ρ(**r**) and s(**r**) reconstruction upon employing either a standard angular mesh for defining the integration grid within atomic β-spheres (a) or a much finer mesh, with one order of magnitude more angular points (b). In general the accuracy in SDD reconstruction is worse than for ρ(**r**). Indeed, when a standard integration recipe is employed (Figure S9a), in most regions |*f*2| is larger than 5 %, with a maximum of ≈ 15% near the H nucleus. This is due to the fact that, at variance with $\nabla^2\rho$, $\nabla^2 s$ is far from being spherically symmetric in the atomic cores. Therefore, to reconstruct adequately s(**r**), a very accurate angular mesh is required also to integrate zones far from the basin boundaries and well within the β sphere. By using a denser angular net (Figure S9b), the accuracy can be significantly improved, with |*f*2| < 1 % for almost the whole *t* interval. The largest error concerns just a single point where *f*2 = –4.3 % at *t* = 0.56. The SDD is quite low at that point ($1.5\cdot10^{-3}$ a.u.), as close to change sign from positive to negative. For the sake of comparison, the currently accepted lower limit to accurately reconstruct the ρ(**r**) scalar through the SF analysis is 10^{-3} a.u..[4] All the integrations discussed in this work were performed through the finer grid.

REFERENCES

(1) M. J. Frisch, G. W. Trucks, H. B. Schlegel, G. E. Scuseria, M. A. Robb, J. R. Cheeseman, G. Scalmani, V. Barone et al., Gaussian09, Revision A.1, Gaussian Inc. Wallingford CT, U.S.A., 2009.

(2) Although it would seem logic to obtain the magnetic orbitals from the diagonalization of the α-density matrix (or of the α- and β-density matrices for an anti-ferromagnetic system) rather than from the total density matrix , this procedure appears not viable in most cases. Indeed, if there are occupied orbitals of the same symmetry of a magnetic orbital, these are allowed to mix with that magnetic orbital, because of their equal (1), or mostly equal occupation numbers. Such problem is clearly avoided when the full (α+β) density matrix is instead diagonalized. As a proof of our assertion, we note that for $^3B_1$ H_2O none of the occupied α-orbitals obtained from the diagonalization of the ROHF α-density matrix resembles the A_1 symmetry magnetic MO. Conversely, the A_1 symmetry magnetic natural orbital obtained from the ROHF total density matrix gives exactly the same local and integral electron density and electron spin density contributions as those obtained from the A_1 symmetry magnetic MO. As a further proof, we note that the very close resemblance we have found between the magnetic contributions derived from ROHF, UHF and CASSCF model wavefunctions is completely lost if magnetic natural orbitals are obtained from the diagonalization of the α-density matrices. Moreover, since all occupied natural orbitals from α-density matrices have occupation number of one (or almost one), the selection of the A_1 symmetry magnetic natural orbital would become fully arbitrary.

(3) F. W. Biegler-König, R. F. W. Bader, ; T.-H. Tang, T.-H., J. Comput. Chem., 1982, 3, 317 – 328. AIMPAC download page:
http://www.chemistry.mcmaster.ca/aimpac/imagemap/imagemap.htm

(4) K. Momma, F. Izumi, J. Appl. Crystallogr., 2011, **44**, 1272–1276.

(5) C. Gatti, D. Lasi, *Faraday Discuss*., 2007, **135**, 55-78.
